# Translating the Elements of Health Governance for Integrated Care from Theory to Practice: A Case Study Approach

**DOI:** 10.5334/ijic.3106

**Published:** 2018-01-31

**Authors:** Caroline Nicholson, Julie Hepworth, Letitia Burridge, John Marley, Claire Jackson

**Affiliations:** 1 Primary Care Clinical Unit, University of Queensland, AU; 2 Mater Research Institute, University of Queensland, AU; 3 Mater Misericordiae Ltd, South Brisbane, AU; 4 School of Public Health and Social Work, Queensland University of Technology, AU; 5 School of Human Services and Social Work, Griffith University, AU; 6 Faculty of Health Sciences, University of Queensland, AU

**Keywords:** integrated care, governance, health, leadership, policy, case study

## Abstract

**Introduction:**

Against a paucity of evidence, a model describing elements of health governance best suited to achieving integrated care internationally was developed. The aim of this study was to explore how health meso-level organisations used, or planned to use, the governance elements.

**Methods:**

A case study design was used to offer two contrasting contexts of health governance. Semi-structured interviews were conducted with participants who held senior governance roles. Data were thematically analysed to identify if the elements of health governance were being used, or intended to be in the future.

**Results:**

While all participants agreed that the ten elements were essential to developing future integrated care, most were not used. Three major themes were identified: (1) organisational versus system focus, (2) leadership and culture, and, (3) community (dis)engagement.

**Discussion:**

Several barriers and enablers to the use of the elements were identified and would require addressing in order to make evidence-based changes.

**Conclusion:**

Despite a clear international policy direction in support of integrated care this study identified a number of significant barriers to its implementation. The study reconfirmed that a focus on all ten elements of health governance is essential to achieve integrated care.

## Introduction

Internationally, countries are reorientating health systems to embrace an integrated, people-centred approach to reorganising health services to improve quality, people’s experience and sustainability [1,2,3]. Challenges faced by health systems, such as ageing populations, the burden of long-term chronic and complex conditions and preventable illnesses requiring multiple interventions over time, demand a fundamental shift in the way health services are funded, managed and delivered [4,5,6,7]. Requisite to meeting these challenges given the involvement of multiple organisations, and therefore varying governance arrangements, is strengthening shared governance, across the local health system where integrated care initiatives are being implemented [1,8,9].

Integrated care is a patient-centred, multi-level, multi-method strategy designed to achieve improved coordination of services across the care continuum of complex health systems [3,10,11]. Integrated care develops within distinct and differing governance structures [12] across three levels of integration – micro, meso and macro [13,14]. At the meso-level of the health system collective action of organisations is required to meet the needs of a population across the care continuum for which they have collective responsibility [13,14,15,16].

In Australia, there were changes at the meso-level from 2011 in regional organisational structures and governance arrangements giving these organisations greater autonomy and accountability for a shared population. Primary health organisations (PHOs), governed and funded by the Commonwealth Government, at the time of the study Medicare Locals, transitioned to Primary Health Networks (PHNs) in July 2015. A key objective of these PHOs was to determine population health need and commission health services to meet these needs for the population within their geographical area focusing on coordination of care by working with other parts of the primary health care system, acute care, social care, community and public health services. In July 2012, health districts, managed locally, governed and funded by the State/Territory health department, became regional Local Hospital Networks (LHN) (named Health and Hospital Services in Queensland) with their own board to manage and oversee the operations to provide public health and hospital services within a given region. With these changes the health governance model for integrated care for newly formed meso-level organisations, collectively responsible for a common geographical population, with mostly common geographical boundaries, some with multiple LHNs to one PHN, was not certain and provided a ‘test bed’ for this study.

Of the modes of meso-level governance described, hierarchy, market and network [13,14,15,17], in the Australian health care setting meso-level governance mechanisms fit more closely with the network form based on relationships and mutual interest rather than a formal structure of authority [13,14,16,17].

There are examples of organisational level integration that have demonstrated improvement in the health of populations, for example, in New Zealand, England and Nordic countries [18,19], and in the United States there are a growing number of accountable health communities [20]. In Australia, there are examples of meso-level integrated care planning and implementation but no published outcomes [21,22], despite many years of policy emphasising the importance of integrating health and social care and, supported geographic regional health integration across the continuum of care, that is, services are provided for all levels and stages of care, [23,24,25]. Most recently, the Primary Health Care Advisory Group, whose role it was to examine opportunities for the reform of primary health care [26] in improving the management of people with complex and chronic disease, recommended better integrated community and acute care via meso-level organisational collaboration [27].

This recommendation was included in the Heads of Agreement between the Commonwealth and the States and Territories on Public Funding which stated that: ‘all governments have a shared responsibility to integrate systems and services’ [28]. This allowed for flexibility in determining the best model of integrated care locally, including health governance arrangements [28]. Given the focus placed on effective health governance to better integrate care, nationally and regionally, how to best arrange governance arrangements to achieve improved population health outcomes is the role of decision makers [9]. In this study these are the Board members of regional meso level organisations.

Health governance refers to the rules governing tasks or functions of providers, users and decision-makers, through which they negotiate, manage conflict, make collective decisions and exert authority [1,9,29,30]. However, a major challenge of health governance to support integrated care is bringing together multiple agencies that are capable of formulating and accepting direction, aligning their efforts to meet the needs of stakeholders and the scale of delivery, agreeing targets to fulfil common goals, and then carrying out their duties [18,19,20,21,31,32,33,34,35].

Problems hampering this form of health governance include ‘misaligned incentives … unintended effects of badly thought through policies, nepotism, incompetence, lack of trust and difficulties with long-term planning’ [p. 331]. There is a dearth of research in this significant area [36], therefore, in line with the WHO’s [2016] ‘Strengthening Health System Governance Better policies, stronger performance’, articulating how meso-level organisations support health governance for integrated care that provides the structure for decision making and policy implementation in a system is the focus of this study.

A health governance model to support integrated care is more relevant to inter-sectoral working and described as ‘soft’ or ‘experimental’ governance’ rather than more formal and rigid forms of legal harmonisation [37,38]. This approach ‘provides a more efficient means of addressing complex health policy problems across overlapping jurisdictions’ and ‘establishes a normative perspective which unifies actors across a number of administrative units’ [38] by more indirect network type forms, relations and processes [39]. The present study builds upon our prior research that identified ten governance elements linked to successful health care integration [36] (see Table 1). While no single framework fits all health systems, the ten elements provide a clear focus for integration initiatives and are adaptable to local conditions and settings. While evidence from the Australian reform environment suggests modest progress in some elements, others remain *ad-hoc* or non-existent [40].

**Table 1:** Elements key to meso level organisations working together [36].

Element	Interventions shown to be effective

**1. Joint planning**	Working together agreements to support joint strategic focus for future work between stakeholders focusing on the continuum of care.
**2. Integrated information communication technology**	Systems designed to support shared clinical exchange, such as, Shared Electronic Health Record, and tools to support systems integration linking clinical processes, outcomes and financial measures.
**3. Change management**	Bilateral support for an agreed change process which is managed locally, and has demonstrated leadership, vision and commitment.
**4. Shared clinical priorities**	Target areas for redesign are agreed and multi-disciplinary pathways across the continuum supported.
**5. Incentives**	Funding mechanisms are provided to strengthen care co-ordination and there are incentives to innovative.
**6. Population focus**	Geographical population health focus.
**7. Measurement – using data as quality improvement tool**	Shared data is used for planning, measurement of utilisation focusing on quality improvement and redesign and a collaborative approach to measuring performance provides transparency across organisational boundaries.
**8. Continuing professional development supporting the value of joint working**	Inter-professional and inter-organisational learning opportunities provide training to support new ways of working and align cultures.
**9. Patient/community engagement**	Involve patients and communities in developing the outcome they want.
**10. Innovation**	Resources are available and innovative models of care are supported.

The aim of this study was to explore the perspectives of Board members of meso level organisations, Primary Health Organisations and Local Hospital Networks, in two geographical regions in one State, in relation to two key research questions:

1. Are the key elements of health governance for integrated care evident in current practice?
2. Do the key elements of health governance for integrated care appear in the planned practice of meso-level organisations?

## Method

An exploratory qualitative research design [41,42] was chosen to address the research question because the phenomenon under examination was largely under-researched. While our previous research led to the development of 10 key elements of health governance (36) for integrated care, the research in this field is limited. As stated by Patton (2002):

In new fields of study where little work has been done, few definitive hypotheses exist and little is known about the nature of the phenomenon, qualitative inquiry is a reasonable beginning point of research (p. 193).

The case study approach systematically collects, organises and analyses data about a case of interest [41]. This method enables a contemporary event to be investigated within its natural context [43], often involving interviews, and, building on previously developed theoretical propositions [36], to guide data collection and analysis.

### Selection of cases and the sampling and recruitment participants

A multiple case study design [43] was used to offer contrasting contexts of governance. To maintain anonymity of the cases, only a limited amount of detail about their contexts can be included.

The definition of a case was based on the following:

Cases are units of analysis. What constitutes a case, or unit of analysis, is usually determined during the design stage and becomes the basis for purposeful sampling in qualitative inquiry (Patton, 2002: p. 447).

Therefore, the cases were selected using maximum variation purposive sampling in order to generate information-rich cases that could provide insights and in-depth understanding rather than generalisations [41]. The sampling criteria were: a) PHNs that were formed as part of the first stage of implementation in 2011; and, b) the cases were located in Queensland, Australia to ensure a similar policy context for LHNs formed under equivalent State legislation. Of the five sites identified, one was excluded due to potential conflict of interest. Of the four remaining sites, the lead researcher selected two, from prior knowledge of the sites and judgment to determine the nature of variation that allowed investigation of information-rich cases that manifested the phenomenon of interest with sufficient intensity without being highly unusual [41]. Variation between sites included urban versus regional/rural geographical location, longevity of Board members, executive staff turnover and history of working together at meso-level.

**Figure 1:**
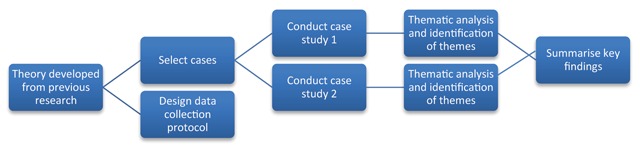
Case study methodology.

Data were not pooled across sites (Figure 1) [43], and cases were treated as distinct units of analysis comprising Board members and Chief Executive Officers (CEOs) of a PHN and LHN in two geographical areas in South East Queensland.

The University of Queensland Behavioural & Social Science Ethical Review Committee granted ethical clearance for this research.

The participants in each case comprised Board members and CEOs of PHNs and LHNs; therefore the age, sex and number were predetermined, namely, thirty-two potential interviewees, nineteen male and thirteen female, across the two selected sites.

Recruitment was by the lead researcher (CN) who contacted the Chairs of PHNs and LHNs at both sites and offered to explain the proposed study at their respective Board meetings. The study was itemised for discussion at Board meetings and Board papers included the systematic review paper [36], together with a Key Participant Information Sheet and Consent Form. Site one PHN and LHN elected to have CN present the research proposal to the Board. Site two elected to have the research key participant information sheet presented at a Board meeting with the option of teleconferencing with CN if questions arose. Following these meetings, the CEOs provided the contact details of members and consent forms were distributed and returned to the lead researcher.

### Data collection and analysis

We used a semi-structured interview schedule because several question areas had already been identified based on the findings of the systematic review [36] (Table 2). Participants were given a choice to be interviewed at their place of work, a LHN/PHN office, the lead researcher’s office or via telephone. The majority of interviews were conducted at the participants’ work place; and the remainder via telephone or at their home. The interviews ranged from 45 to 85 minutes in duration with the majority taking an average of 60 minutes.

**Table 2:** Semi-structured questions asked of each element identified in systematic review [36].

Key research question *for each element*

Is the key element of health governance for integrated care evident in current LHN/PHN practice? – What are the enablers to implementation – What are the barriers to implementation? Does the key element of health governance for integrated care appear in the planned practice of meso-level organisations? How will this element be used in the future?

All participant interviews were conducted by the lead author (CN) between March 2014 and June 2015 at the two case study sites. The interviews were audio-recorded and professionally transcribed verbatim.

To support the construct validity of each case, multiple sources of evidence (open-ended interviews and documentation) were used during data collection to encourage convergent lines of inquiry [43]. While we attempted to obtain documentation supporting LHN/PHN meso-level integrated health governance only one joint regional plan was made available.

The systematic qualitative data analysis was conducted in three phases. First, two authors (CN, LB) independently inductively coded [44] the data manually using qualitative content analysis [45]. Following this the researchers held regular meetings to compare their respective identification of coding units. Second, major themes were identified from the data using thematic analysis involving familiarisation, identification of thematic framework, indexing, charting and interpretation [46]. The coding units, themes and sub-themes were presented to the research team at several meetings where the process for reaching the main themes was discussed and justified. Third, the research team met regularly to further examine and progress the data analysis through discussion of the identification of coding units [45] and develop consensus about the final themes and sub-themes by referring back to the original data.

## Results

Three major themes, and corresponding sub themes, were identified from the qualitative analysis (Table 3) and are presented below and illustrated by selected interview extracts. The extracts include identification of the case it refers to, the participant number, and gender. For example, [C1, P11, m] indicates [Case study 1, participant 11, male]. Each theme refers to current and/or future implementation of integrated governance across the health system, as well as the major enablers and barriers to this practice.

**Table 3:** Thematic framework and subthemes identified.

Major themes title	Focus	Q1: How is the element supported, or not, in the current practice?		Q2: How will/could this element be used in the future?	a. What are key enablers?	b. What are key barriers?
					
		Supports (✓)	Does not support (×)			

1. Organisational versus system focus	Structures support an organisational not system focus		× No system accountability × Funding method prescriptive × Planning not strategic × No team across the continuum × Lack of innovation and focus on the process of change	✓ Accountability for outcomes, joint key performance indicators (KPIs) ✓ Funding reform to allow flexibility and change ✓ Vision for a health system and long term strategy agreed ✓ Focus on care for the population and care across the continuum based on needs	+ Patient-focused care + Change supported, measured and evidence provided	– Short term strategy & policy cycles – Drivers - financial, political, and cultural - not aligned – No joint accountability for population health planning, performance or outcomes
	Access to quality and useful data across the system is essential		× Poor data quality × Data rich, information poor	✓ One central national repository for all data ✓ Needs to be broken down into geographical areas for use locally ✓ Data governance agreed	+ Sharing data across the continuum is key	– Lack of access to quality data – Legal issues – who owns the data, political risk, consent and privacy – Cost

2. Leadership and culture	Leadership skills to develop a ‘system’ approach is essential	✓ Goodwill at executive level ✓ See the need for change	× Lack of leadership, trust and commitment	✓ Boards have to operate in honest and transparent environment and value working in partnership ✓ Board’s commitment demonstrated with joint MOU to support structural alignment	+ Board agreement on common purpose + Determine priorities + Dedicated resources to facilitate under CEO direction	– Lack of leadership and commitment to change – No central co-ordination at government level
	Clinician engagement across the continuum is key	✓ Roles working across the continuum have brought change	× Lacking at senior level × Inadequate resources to support engagement	✓ Clinician leaders identified and supported to lead the way ✓ Use of boundary spanners	+ Clinician leadership - joint clinical governance board to agree protocols across the continuum + Facilitate communication, build goodwill	– Overcoming vested interests to keep things the way they are – Clinician leaders risk-adverse rather than allowed to be sensible risk takers
	Cultural barriers exist		× Risk-averse rather than risk-aware × Perceptions hospitals have the most to gain	✓ Value working together, mutual respect and understanding articulated throughout the sectors	+ Build relationships and professional respect	– Decades of bureaucratic control to overcome – ‘Master/servant’ relationship – Lack of communication and collaboration across the system before decisions are made
	Workforce capacity building is needed		× Seen as operational not strategic	✓ Support interprofessional learning opportunities ✓ Need a driver tasked with this – boundary spanner	+ Shared KPIs for outcomes + Requires strategic support + Requires strategic support	– How do we educate across the continuum? No KPIs for this

3. Community (dis) engagement	Overcoming perceptions		× Not using the community × Preconceived ideas	✓ Need to bring the community on the journey	+ Agreed mandate for engagement across the system	– Perceptions hospital care is best – How do we educate across the continuum? No KPIs for this
	Requires greater priority		× Not a priority	✓ Need a vision to keep people well, not focus on illness	+ Policy directive + Requires designated resources	– Lack of focus on this at Board and Executive level

### Theme 1: Organisational versus System Focus

#### Current practice

Participants reported that the elements were unsupported in current practice. First, at the time of the study, the political and operating environment as meso-level organisations were in the forming stage the focus was on the organisation rather than integrated care at the health system level that served as a major limitation to achieving joint outcomes. The dominant organisational focus operated in several ways. Each organisation was accountable for different key performance indicators (KPIs) and having different priorities. Consequently, the lack of accountability for system outcomes continued to perpetuate organisations’ practices that were siloed, and, were ‘almost competitive, not quite combative’ [C1, P11, m] rather than supporting partnerships.

Additionally, current funders were ‘very prescriptive in what they expect to happen with the funds’ [C1, P1, m]. Working together was seen as being bound by the current rules ‘to work within what we are funded to do’ [C2, P23, m] rather than attempting to trial alternative funding models which were ‘seen as being outside our scope’ [C2, P19, m]. Despite the willingness of some Board members and CEOs to consider alternative funding models, others believed that, without a supporting policy lever, alternative funding models would not be achievable.

While joint planning occurred, it was ‘largely reactionary’ [C1, P3, m] and had taken place organisationally and, not strategically as ‘people think about their own system, their own small subset of a system’ [C1, P16, m]. In relation to shared clinical priorities and care across the continuum, participants reported that ‘no really transparent conversation’ [C2, P19, f] had occurred, and the ‘concept of working together as a team … is a long way from reality’ [C1, P16, m]. These practices were further compounded by the lack of shared responsibility for outcomes.

Innovation was perceived as a ‘secondary order thing’ [C1, P15, m] and there was a lack of strategy and changes were reactive, ‘not strategic’ [C1, P9, f]. Commitment to change was limited with no ‘dedicated time and effort overtly to … managing the change’ [C2, P20, f] resulting in a feeling of constraint working within the current system. Therefore, a lack of strategy for change and commitment with supporting resources were major reasons why participants believed change did not succeed.

Importantly, the second focus area, access to data for effective planning was highly problematic. Current practice precluded access to system-wide data. Existing data lacked quality and was not collected and ‘collated in a meaningful way’ [C2, P29, f]. The effect was to be ‘data rich and information poor’ [C1, P15, m]. Importantly, one party’s willingness to share available data could be hampered by another’s reluctance:

… they won’t give us their data so we’ve had to work independently of them getting data sources from other areas – at times people say ’yes, you can have the data, but that hasn’t transpired [C1, P12, m].

#### Future practice

Using the elements in the future to support a systems approach would require ‘a policy framework’ [C2, P18, f] focused on sustaining a health system which ‘would take a government with some courage’ [C1, P11, m] to action. Also needed were ‘agreed joint KPIs’ [C2, P20, f] aligned with joint strategic priorities, and ‘worked on overtly rather than conveniently’ [C2, P20, f], as well as ‘an agreed joint vision for a system’ [C1, P16, m]. In addition, a focus on partnering was needed to achieve ‘high level population health planning’ [C1, P9, f] and a flexible funding model to support system innovation.

Participants made three suggestions regarding the future availability of data including, First, that there should be access to a national central data repository allowing ‘everyone to use the same data … [which] is accurate … [and] across agency’ [C2, P19, f]. Second, joint data analysis should occur to develop a population plan that is meaningful locally. Third, these activities need be carried out with an agreed data governance protocol in place between stakeholders.

#### Enablers and barriers

Several enablers of a system-focused approach were identified: a shared vision; measuring outcomes for populations; shared accountability; clinicians’ involved in determining priorities; support for change; and shared data, because ‘without sharing data you’ll never get the evidence’ [C1, P6, f]. There was a clear focus on getting ‘as much value as we can out of the health dollar’ [C2, P28, m] and changing the funding model to support shared priorities and shared benefit was seen as a way of promoting integrated care.

However, there were also significant barriers identified by participants. The KPIs of different sectors within the system were seen as supporting ‘fractured jurisdictions’ [C1, P2, m]. There seemed to be no long-term strategy for the health system and, despite the rhetoric, the political will to truly integrate the system was lacking. Lack of access to timely, meaningful, useful data was a major barrier to determining joint priorities and measuring outcomes. The current funding system was seen as inefficient and supporting competitiveness with there being no ‘ability to change that’ [C2, P20, f].

### Theme 2: Leadership and Culture

#### Current practice

Leadership, a fundamental requirement for system level strategy development and change, was reported to be significantly lacking in some areas at Board and Executive levels. One participant noted the current Board’s lack of enthusiasm and direction to enable progress in working together due to lack of clarity ‘about what it aims to do’ [C1, P4, f]. Leaders were needed who recognised the imperative for change and who would support it as a key strategy rather than implementing and imposing existing policies. The interorganisational relationship between Boards and CEOs was crucial to working effectively together, and was perceived as requiring a ‘lot of goodwill’ [C1, P15, m], and trust and respect which may not exist. It was apparent that the ‘knowledge sharing and the maturity of both sides is not quite there yet’ [C1, P7, f]. Finally, participants felt at ‘political levels, the rhetoric is there but not the leadership’ [C 2, P18, f]. The current system supports fragmentation, is largely reactive and vision is lacking, both organisationally and politically, for the system to drive strategy and change.

Participants strongly supported senior clinician leadership and engagement as being key to developing care models across the continuum, but such activity was perceived as ‘sporadic’ [C2, P23, m] and also lacking. In some cases where initiatives had brought stakeholders together, there had been ‘recalcitrant clinicians within the hospital system and primary care’ [C1, P2, m] who had different agendas and ‘a bit of a turf war’ [C1, P9, f] resulting in engagement being hampered by entrenched silos, and lack of relationships and trust.

Indeed, an adversarial culture was identified that was typified by ‘a master/servant relationship’ [C1, P12, m] between organisations:

“… the hospital sector has to drive it because I think we’re the ones with the most to gain … Very happy if the primary health care sector comes up with good ideas and shows a willingness, … we have to tell the primary health care sector what’s important and what’s not important and what they can do to help us” [C1, P17, m].

Unfortunately, ‘decades of centralised bureaucratic control’ [C2, P21, m] resulting in lack of trust and a culture of ‘risk aversion rather than risk awareness’ [C2, P18, f] have reduced the ability to innovate, since ‘some [clinicians] are willing and then the culture sort of blocks any initiative that they have’ [C1, P12, m]. Most participants identified existing entrenched cultures as a major constraint in developing an integrated health system.

Finally, workforce capacity building via interprofessional learning across the continuum was seen as an organisational and operational issue, not strategic. The current system supported ‘a bunch of two-way relationships developing on disparate fronts’ [C1, P3, m] and interprofessional, interorganisational education was seen as a ‘luxury item’ [C1, P2, m], with no shared key performance indicators for accountability to support it and where ‘interdisciplinary head-butting’ [C1, P7, f] and a lack of understanding persist.

#### Future practice

Future leadership, at Board, CEO and political levels, needs ‘tenacity’ [C1, P16, m], to articulate a vision for the system so that there are ‘shared goals and everybody is on the one page’ [C1, P13, m], with a readiness for risk-taking and innovation, and, support for the value of working together.

Backed by an integrated system-wide strategy, senior clinicians need time to undertake this work across the continuum. Key to implementing these changes and engaging people is someone who can work across organisations and professions focusing on mutual gains and outcomes being ‘beneficial at both ends’ [C1, P7, f].

The culture of organisations needs to demonstrate ‘mutual respect and understanding … to make those systems work’ [C1, P9, f]. As this happens, ‘if you’ve got that culture of success then the next thing is, what can we do next to innovate’ [C2, P30, f]. Nurturing this culture of collaboration and joint outcomes can only occur if it is supported from leaders within organisations.

Interprofessional and interorganisational education was seen as a way of supporting clinicians to increase knowledge about jointly working together, ‘learning that you’re one piece of a very big pie’ [C2, P29, f] and identifying new ways of working. Interprofessional interorganisational education can help break down misperceptions and support care across the continuum.

#### Enablers and barriers

The most significant enabler was leaders who could set and drive an agreed strategy. The lack of a clear policy direction presents a void that participants believed Boards could fill by agreeing a common purpose and shared vision; setting ‘mutually beneficial shared goals’ [C1, P1, m] with accountability for outcomes; an articulated need and allocation of resources for change and a willingness to work together; and supporting innovation to achieve outcomes. Participants noted a key component was ‘allowing time to develop good relationships’ [C1, P10, m] at Board level to support a working environment built on trust, commitment, professional respect and knowledge. A strategy to implement change was to support clinician leaders in determining priorities and having accountability for outcomes, ‘it’s all about KPIs and that will change the culture’ [C1, P12, m]. However, it was also noted that engaging clinicians as leaders is a ‘long and drawn out process’ [C2, P22, m].

The main barrier currently was the focus on short-term political gains and not on long-term solutions or potential solutions. Participants acknowledged that commitment to change had been difficult in this environment and that survival of their own organisation was their key focus. The lack of policy direction, a funding model supporting care in silos and who bears the risk for joint initiatives meant ‘both boards are clearly aware of the problems, it’s just that they have worked on their solutions somewhat independently … the solutions are not shared’ [C1, P16, m]. Another barrier was fear keeping clinicians and executives in their silos; overcoming years of entrenched behaviours and ‘thinking outside the square, doing something that hasn’t been done before … the freedom to do that hasn’t been there’ [C2,P21, m]. Clinician leaders had been hampered by lack of dedicated time to change how they provide care, and in some cases, there were ‘vested interests in keeping everything just the way it is’ [C1, P16, m]. There was enthusiasm to overcome some of these barriers and some progress but not the policy to underpin and support such changes to occur at the system level.

### Theme 3: Community (dis)Engagement

#### Current practice

The third major theme revolved around community engagement. While individual organisations had some form of community engagement strategy the practice of engaging communities as partners was clearly lacking from a system perspective. Participants’ believed that ‘the community still expects largely to be told what’s going to happen to them, by whom and when’ [C1, P2, m] rather than be included as a vital part of decision making. There was also a perception that the community felt that hospitals were ‘a safe place for them to be’ [C2, P29, f] although this was not based on any organisational evidence but rather ‘anecdotal-type stuff’ [C1, P1, m]. Community engagement was ‘not seen as a priority’ [C2, P26, m], they had ‘forgotten that step that we should be taking our community with us’ [C2, P23, m] and that ‘sometimes I think we pay the lip service to that’ [C1, P3, m]. Participants perceived that engaging communities was an area that needed considerable work and development.

#### Future practice

In the future participants suggested a process was needed that enabled community engagement from a system perspective which needed to articulate ‘a philosophy or vision of how we keep people well … as a starting point’ [C2, P29, f], focusing on wellness not illness. As one participant noted; ‘I do think it’s possible for the consumers to own more of their healthcare than we give them both credit for and ability to do’ [C2, P18, f].

#### Enablers and barriers

The major enabler to facilitate community engagement was perceived to be a joint agreed clear mandate for community engagement across the system. A State policy directive determined key performance indicators for hospitals which ‘made very clear we must engage our community’ [C2, P18, f], but from a system perspective there was no joint accountability. Commitment to community engagement should include ‘having the right people within the organisation … to engage with these people and identify the stakeholder lists’ [C1, P1, m]. This required a joint commitment to resourcing and designated people to drive it.

The major barrier was addressing community expectations; that people have been conditioned to go to hospital when ill and perceive a ‘community based service isn’t as good as … the hospital even though it might be more appropriate’ [C1, P10, m]. Getting community buy in to the process of change and keeping them informed was seen as an issue. Although one participant stated that ‘a dangerous offshoot … would be the community thinking … they don’t know what they are doing because they keep asking us’ [C1, P2 m] the majority asked for a far greater and inclusive engagement strategy. Finally, the lack of attention to community engagement by leaders that there was no accountability for this and it tended ‘to go to the bottom’ [C2, P30, f].

## Discussion

This is the first study, tested in two regions in Queensland, Australia, to systematically examine the current and anticipated future use of the governance elements [36] that underpin successful health care integration between meso-level organisations. Most elements were not currently used by meso level organisations to develop health care, although participants agreed they were essential. This highlights the challenges of implementing higher-level change based on evidence indicating that all ten elements are fundamental to successful and sustainable integrated care, particularly for patients at risk of poor health outcomes or with chronic and complex health needs [47]. The findings yielded three key insights into the challenges of implementing the elements.

First, an organisational rather than system approach was seen to significantly impede the development and implementation of system-focused reform. Participants’ views concurred with others’ [48] who argue for a ‘system of care’, and who highlight policy barriers and legislation that pull the system away from collaboration [6,49]. A ‘whole-of-system’ approach was lacking in the present case study, including the pooling of local population data, integral to understanding needs, targeting strategies and monitoring outcomes [19]. Overcoming real or perceived barriers to data sharing between organisations precedes any gains [19] and participants regarded lack of access to quality system-level data as a major impediment to developing a joint population-level plan.

Second, leadership and culture influenced success in care integration. This study is consistent with existing literature in that leadership was lacking at multiple levels [50] of the health system. At political level leadership is essential to remove policy barriers that inhibit joint working [49] and promote a lack of both co-ordination and collaboration [6]. Meso level leadership to facilitate collective decision-making between organisations [49] or trust were evident in this study. Participants held organisational responsibility without formal authority to make decisions on behalf of the system; and leadership styles that facilitate agreement and consensus were lacking [49]. Participants acknowledged the need for future leadership to implement change through ‘soft power’, recognise the need to work across boundaries and build relationships and enable organisations to develop cultures that support collaboration within the system [50]. Such leaders of transformation would guide rather than control [51] and be willing to ‘give away ownership’ [50]. The present study found the issue of system leadership was a significant barrier to achieving integrated care and supports previous research which recognises ‘that effective system leadership is a necessary prerequisite to achieving truly effective care co-ordination’ [52].

This study acknowledged the requirement for significant clinician leadership from across the system that could influence colleagues [6] and support care across the continuum. Whilst participants reported the support and skills to do this existed in the current workforce implementation was sporadic due to lack of relationships and trust [53]. The present study found that, despite evidence supporting workforce capacity building, using shared clinical pathways and education across the continuum to facilitate multi-disciplinary teamwork [53,54], developing interprofessional respect and breaking down interorganisational and interprofessional cultural perceptions [55] had not been identified as an opportunity at a strategic level.

Third, in this study community engagement in healthcare planning processes was markedly under-developed, and hence, termed community (dis)engagement. There was a lack of strategy to engage with ad hoc action plans for working with individual organisations, and a perception that patients regarded hospital as the optimal form of care. Patient and community involvement have also been missing in planning processes [36] despite evidence that the focus has shifted for consumers to participate not only in their health but also in innovation and value co-creation in health care [56].

## Limitations

This research included only two cases sampled from one State, although political and economic contexts of health care planning across Australian regions and States and other international contexts are diverse. Therefore, the transferability of the findings to other contexts of heath care planning is limited. However, the findings do demonstrate several important dynamics of health care leadership and planning that are internationally required to support a governance model that could facilitate integrated primary/acute care. The study was also undertaken at the time of an emerging period of change in the Australian context with Medicare Locals transforming to Primary Health Networks causing significant disruption within the health sector. Finally, despite every attempt to maintain anonymity of case study areas, the participants’ prominent positions in the local health care system may have affected what they were willing or permitted to disclose during interview.

## Conclusion

As the burden of complex chronic disease increases requiring health systems to be people-centred and better meet patient needs, internationally, there is a growing focus on integrated care. Underpinned by clear evidence, partnerships, shared responsibility and joint working between meso level organisations in different sectors can create systems that are capable of transforming care. However despite policy directions in Australia supporting integrated care and a mandate to strengthen and promote collaboration this research has identified there are significant barriers to overcome in some meso level organisations. In building future health systems, organisations need to address the lack of aligned system drivers, the need for robust and high quality data, more effective leadership at executive and clinician levels, and the significant lack of community engagement. Finally, the study reconfirmed that a focus on all ten elements for integrated governance is agreed as being essential to achieve integrated care.
